# Research on prediction of residual deformation in goaf of steeply inclined extra–thick coal seam

**DOI:** 10.1371/journal.pone.0240428

**Published:** 2020-10-16

**Authors:** Changfu Huang, Qun Li, Shuguang Tian

**Affiliations:** 1 China Railway 15th Bureau Group Co., Ltd., Shanghai, China; 2 College of Mining Engineering, North China University of Science and Technology, Tangshan, China; University of Science and Technology Beijing, CHINA

## Abstract

The residual deformation of a goaf is studied to improve the foundation stability assessment for metro lines passing through the subsidence area of steeply inclined extra-thick coal seams. The variable mining influence propagation angle is introduced to describe the special form of the rock movement. Based on the modified parameters in the traditional probability integral model, a subsidence prediction model is established. Then, based on the idea of an equivalent mining thickness, Kelvin model is introduced to analyze the creep characteristics of the old goaf, and the dynamic prediction function of the residual subsidence is constructed to realize the dynamic analysis of the residual deformation. Moreover, a case study is used to evaluate the predictive effectiveness of the prediction model, and the results are compared with the monitoring data and numerical simulation results. The results show that the values with a relative error between the predicted value and measured value are in the range of ±7%, indicating that the prediction model based on the mining influence propagation angle is feasible. Thus, the residual deformation prediction model based on the mining influence propagation angle is considered to be suitable for predicting the subsidence of engineering projects crossing a goaf.

## Introduction

With the rapid development of urban rail systems and the continuous construction of mining areas in China, some new metro lines will inevitably pass through an old goaf. Although the old goaf has experienced long-term natural compaction, it is still possible that the broken rock mass gap is not compacted. The relative stress balance in the broken rock mass is likely to be broken under the influence of the external environment, such as construction. The secondary deformation in the collapsed area can occur, and then the metro structure will be destroyed by the larger residual deformation above the goaf. Therefore, the prediction of residual movement and deformation is very important for the construction of metro structures above old goafs.

When constructing a metro above an old goaf, the evaluation of the foundation stability is a key issue that must be studied, and residual deformation is the main factor affecting foundation stability. Many scholars have constructed the corresponding prediction model for the residual deformation of a goaf. Zhu et al. analyzed the evolution of the overburden structure in a goaf, and defined two deformational phases of the surface residual movement. The detrimental impact on the buildings above a goaf is the residual deformation of the second stage [1]. Guo et al. established the random medium model to analyze the residual subsidence of a long–wall goaf on the basis of the activation mechanism of fractured rock in a long–wall goaf. The model was introduced for practical cases to verify the accuracy of the prediction results of the residual subsidence [2]. Deng et al. argued that the residual subsidence of the old goaf is mainly caused by mining fractured rock voids, and established a calculation method for the residual subsidence coefficient on the basis of the stress-strain relationship of the fractured rock mass [3]. Wang et al. established the residual subsidence prediction model of a long-wall old goaf after introducing the residual subsidence coefficient, and predicted the surface residual subsidence of a horizontal old goaf [4]. Wang et al. proposed the multivariable discrete gray prediction model for the residual subsidence, which solves the obvious fluctuation of the residual subsidence series and poor prediction with traditional grey models in the old goaf [5]. Dai et al. established a prediction model of the surface movement caused by extra-thick coal seam horizontal slicing mining based on the deformation mechanism of overlying strata in steeply inclined coal seam mining and the variation characteristics of the propagation direction of the surface movement [6]. Díaz-Fernández et al. presented a computer tool that automatically predicted mining subsidence using the generalized n-k-g influence function [7]. Cowie et al. presented methodologies using gravity anomaly inversion, residual depth anomaly (RDA) analysis and subsidence analysis, and applied this method to the west Iberian rifted continental margin [8]. Li et al. introduced the FPM method for the prediction of surface subsidence due to inclined coal seam mining [9]. Nummedal et al. found that the new residual subsidence data permit rigorous testing of existing geodynamic models, and revealed that the residual subsidence is of a dynamic topographic nature [10]. Bau et al. developed an original nonlinear three-dimensional finite element model to predict the residual land subsidence adjacent to a depleted gas field [11]. Hu et al. pointed out that the residual deformation is caused by the compaction of the cavity cracks in the fracture zone of the collapsed area, and the calculated length of the goaf after long-term development is equal to the mining length. Based on this hypothesis, an equation for predicting residual deformation was proposed, and the reliability of the equation was verified [12].

However, the existing research was on the study of the residual deformation of thin horizontal coal seams or gently inclined coal seams in long-wall mining or on the study of the surface deformation caused by the mining of steeply inclined extra–thick seams [13]. There is little research on the prediction of the residual deformation for the old goaf in steeply inclined extra-thick coal seams. The purpose of the present paper is to construct a prediction model to analyze the residual deformation in the steeply inclined extra-thick coal seam. Therefore, the remainder of the paper is organized as follows. First, the residual deformation mechanism of the goaf in the steeply inclined extra-thick coal seam is analyzed. Second, a residual deformation prediction model based on the mining influence on the propagation angle is constructed, and Kelvin model is introduced to construct a dynamic prediction function for residual deformation. Finally, the validity of the prediction model is evaluated in comparison with the measured data and numerical simulation.

## Mechanism of residual deformation in the goaf of a steeply inclined extra-thick coal seam

**Fig 1:**
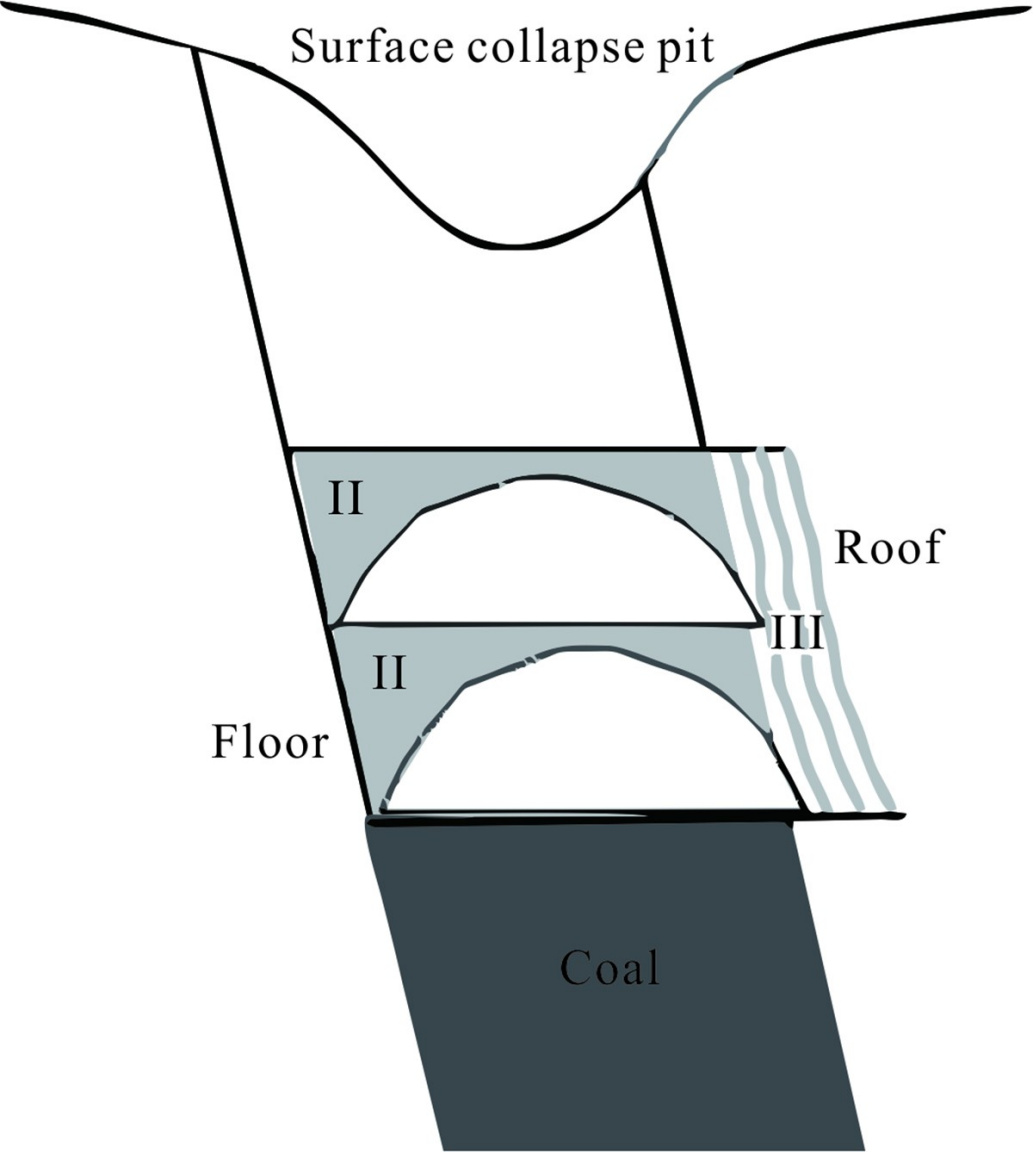
Destruction zone of the top coal and surrounding rock.

**Fig 2:**
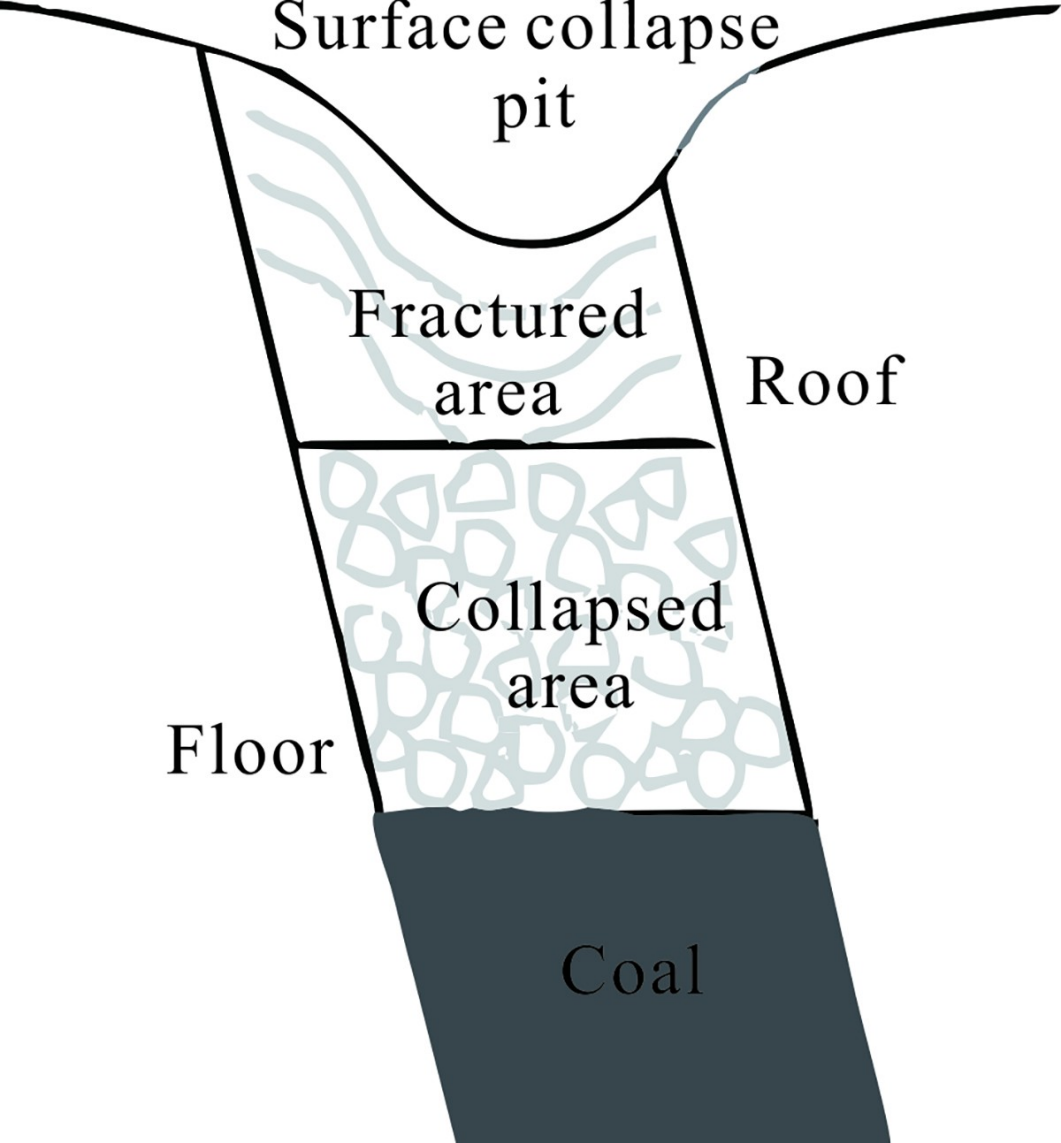
Zoning situation after the goaf collapse.

The surface deformation caused by mining can be divided into two stages. The first stage is the deformation of the overburden rock during the mining process and the surface deformation within 3~5 years after mining, which is called the large deformation stage. The mining regulation notes that the surface is stable after the large deformation stage [14]. The second stage, called the small deformation stage, is the residual deformation stage after surface stabilization. The details of these two stages are as follows:

(*i*) *Large deformation stage* The destruction process of the top coal and surrounding rock due to mining can be generalized into three regions, as shown in Fig 1. Region I is the arch area formed by the destruction of the top coal, which is caused by horizontal sublevel mining. When the original stress equilibrium state of the rock mass around the goaf is destroyed, the direct top coal is first broken and then successively collapses. The upward development causes the collapse of the overlying coal, thereby causing surface movement and deformation. Region II is the floor sliding area. Part of the top coal near the floor slides down along the coal floor and fills the goaf after mining. Region III is the roof separation area. Along with continuous mining, the roof rock is destroyed after being exposed to a certain degree, and it develops in the direction of the goaf. With the continuous crushing of the top coal and the upward development of the floor sliding area, the caving of overlying rock gradually developed to the surface, and then a collapse pit on the surface was formed.
(*ii*) *Small deformation stage* The continuous surface deformation time is 3~5 years, that is, it starts from a surface subsidence of 10 mm and continues until it sinks no more than 30 mm in 6 consecutive months [14]. After continuous surface movement, the goaf and overlying rock basically form two relatively stable areas, the collapsed area and fractured area. These stable areas can be seen in Fig 2. The collapsed area is composed of a broken roof and the overlying fractured rock mass, and it is quakily filled by the collapsed body due to the expansibility of the collapsed body. The fractured area is composed of an overlying fractured rock mass and a surface subsidence rock mass. The goaf is filled with collapsed overlying rock masses, which are in a broken and discrete state. Under the influence of the external environment (such as earthquakes, metro construction, etc.), the incompletely compacted cracks and separation layers may destroy the relative stability of the overlying structure. The old goaf can be activated, causing new movement and deformation to appear on the surface, called residual deformation. The residual deformation can be more obvious when mining in steeply inclined extra-thick coal seams. In this paper, the activated cavities and cracks in the goaf are converted into an equivalent mining thickness to predict the residual deformation.

## Residual deformation prediction model based on the mining influence propagation angle

The probability integral method is a prediction model for the mining subsidence of horizontal layered ore bodies, which is based on isotropic or horizontal isotropic rock masses. The method has been widely used in studies of surface subsidence caused by the excavation of horizontal or gently inclined seams. Nevertheless, these studies, on the basis of the traditional random medium theory, qualitatively describe the propagation path from the goaf to the overlying rock mass. In fact, with the increase in the dip angle of the coal seam, the degree of anisotropy in the original isotropic layer will continue to intensify, which causes the traditional processing method to produce a large deviation. Therefore, there is a certain defect in the analysis of surface subsidence using the traditional probability integral method for steeply inclined extra-thick coal seams.

For the mining of steeply inclined extra-thick coal seams, the shape of the subsidence basin does not appear as a symmetrical vertical normal distribution curve like the mining of a horizontal layered ore body, but it shows obvious asymmetry. The direction of the rock movement near the floor is mainly in the form of shear slippage, and the destruction of the separation near the roof spreads to the collapsed area. The two propagation directions are different, which demonstrates the nonuniformity of the moving direction of the rock in the steeply inclined extra-thick coal seam; that is, the mining influence propagation angle is not a fixed value but a variable. Therefore, this paper adopts the variable mining influence propagation angle to describe the special form of rock movement. The subsidence prediction model based on the changing mining influence propagation angle is constructed by modifying a certain parameter in the traditional probability integral model.

### Mining subsidence prediction model based on the mining influence propagation angle

**Fig 3:**
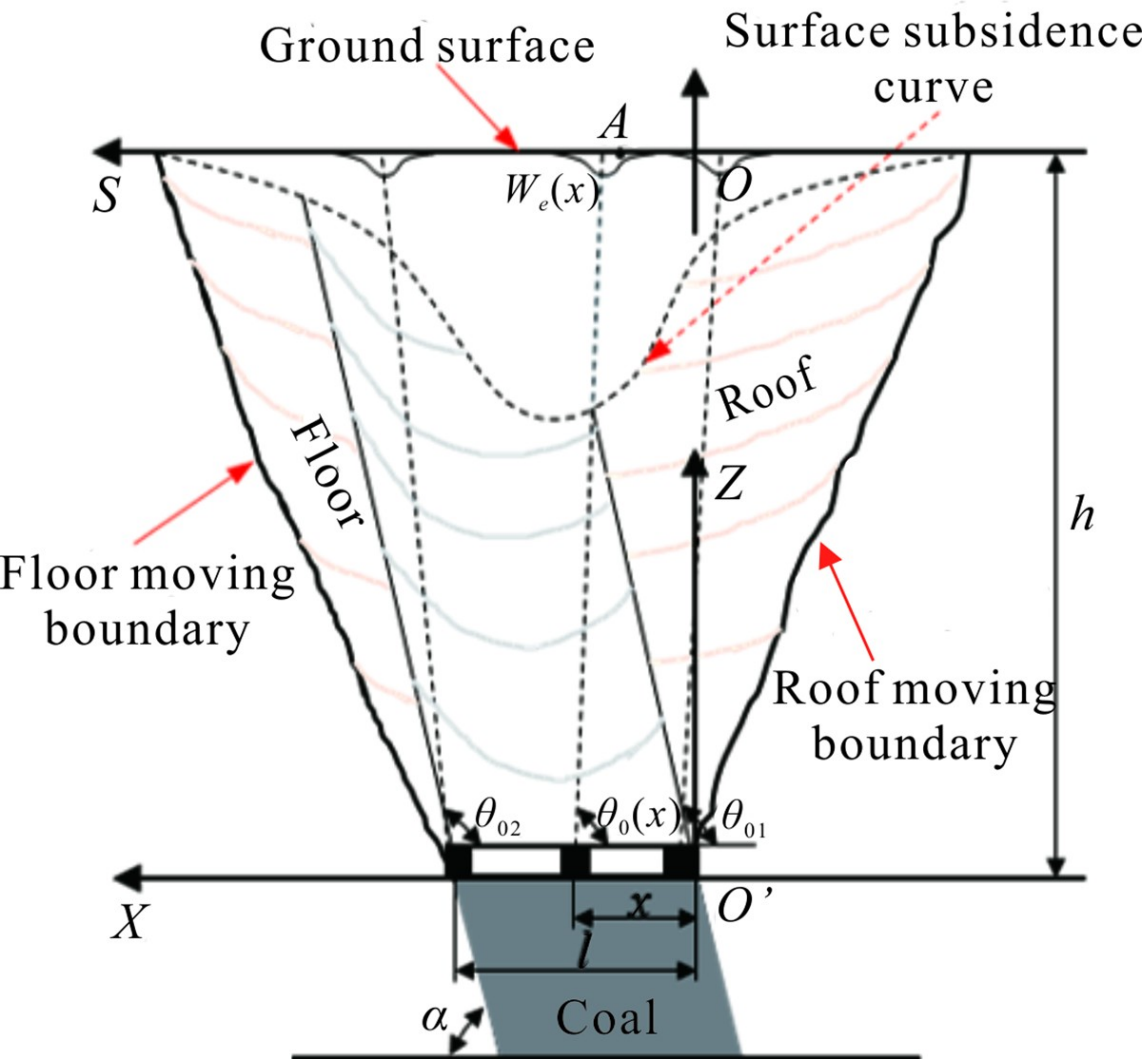
Surface subsidence of a unit in the horizontal section top coal caving of the steeply inclined extra-thick coal seam.

Fig 3 presents the surface subsidence curve due to horizontal section top-coal caving in the steeply inclined extra-thick coal seam. As shown in Fig 3, the coordinate system *XO*’*Z* is established, the symbol *α* represents the dip angle of the coal seam, and the symbol *h* represents the mining depth. The sublevel working surface is divided into the mining unit along the horizontal direction, the length of each horizontal working face is *l*, and the horizontal ordinate of the mining unit is *x*. The mining influence propagation angle *θ*_0_, which is a non–fixed value, is a function of *α* and *x* and can be expressed as *θ*_0_(*α*,*x*).

In Fig 3, the mining influence propagation angle *θ*_01_ near the roof and the mining influence propagation angle *θ*_02_ near the floor are the minimum and maximum values of the mining influence propagation angle of the mining unit, respectively. The mining influence propagation angle *θ*_0_(*x*) of the intermediate mining unit changes with the horizontal ordinate *x* of the mining position. The mining influence propagation angle *θ*_0_(*x*) of the horizontal arbitrary mining unit can be obtained according to the linear interpolation method [6].

$$\theta_{0}(x)=\theta_{01}+\frac{x}{l}(\theta_{02}-\theta_{01})$$ (1)

For steep seam (*α*>45°) mining, the mining influence propagation angle can be expressed as follows [15].

$$\theta_{0}=90{}^{\circ}-k_{0}(90{}^{\circ}-\alpha ),k_{0}=\{\begin{array}{c} 0.5∼0.6(\mathrm{soft}\;\mathrm{rock})\\ 0.6∼0.7(\mathrm{neutral}\;\mathrm{rock})\\ 0.7∼0.8(\mathrm{hard}\;\mathrm{rock}) \end{array}$$ (2)

where *k*_0_ is the coefficient of the mining influence propagation angle, and the range of the values is generally [-0.2, 0.2] for steeply inclined extra-thick coal with a soft floor.

Then, *θ*_01_ and *θ*_02_ can be obtained.

$$\{\begin{array}{c} \theta_{01}=90{}^{\circ}-k_{1}(90{}^{\circ}-\alpha )\\ \theta_{02}=90{}^{\circ}-k_{2}(90{}^{\circ}-\alpha ) \end{array}$$ (3)

where, *k*_1_ and *k*_2_ are the coefficients of the mining influence propagation angle.

Substituting Eq (3) into Eq (1) yields the mining influence propagation angle of the horizontal arbitrary mining unit.

$$\theta_{0}(\alpha ,x)=90{}^{\circ}-[k_{1}+\frac{x}{l}(k_{2}-k_{1})](90{}^{\circ}-\alpha )$$ (4)

which reflects the change mechanism of the rock layer movement and propagation direction in the steeply inclined extra-thick coal seam with horizontal mining.

The expression of the unit mining subsidence basin is as follows.

$$W_{e}(x,z)=\frac{1}{r_{z}}\exp\{-\frac{\pi {\left\{x-z\cot\{90{}^{\circ}-[k_{1}+\frac{x}{l}(k_{2}-k_{1})](90{}^{\circ}-\alpha )\}\right\}}^{2}}{r_{z}^{2}}\}$$ (5)

where *W*_*e*_(*x*,*z*) is the subsidence value of point (*x*, *z*) caused by unit mining; *r*_*z*_ is the main influence radius of the subsidence basin where the depth of the overlying rock from the working face is *z*.

For the whole unit thickness seam mining, the subsidence value *W*_*u*_(*s*) of surface point A with horizontal coordinate *s*, is the sum of the subsidence values caused by each unit mining in the range of 0 to *l*.

$$W_{e}(s)=\int_{0}^{l}\frac{1}{r}\exp\{-\frac{\pi {\left[x-s-h\cot\theta_{0}(\alpha ,x)\right]}^{2}}{r^{2}}\}dx$$ (6)

If the slicing thickness is *m*, the subsidence value *W*(*s*) of surface point A can be written as follows.

$$W(s)=mq\cos\alpha^{'}\int_{0}^{l}\frac{1}{r}\exp\{-\frac{\pi {\left[x-s-h\cot\theta_{0}(\alpha ,x)\right]}^{2}}{r^{2}}\}dx$$ (7)

where *q* is the subsidence coefficient of slicing mining in the large deformation stage; *α*' is the dip angle of the slicing working face, where *α*' = 0 when the horizontal slicing method is adopted.

### Residual deformation prediction model based on the mining influence propagation angle

The residual deformation of the goaf in the steeply inclined extra-thick coal seam resulted from the compaction of the collapsed area during the small deformation stage. The goaf does not form a boundary zone and an intermediate zone but forms a whole collapsed area, that is, the inflection point offset is *S*_0_ = 0. When calculated by the probability integral method [16], the calculated length is equal to the length of the horizontal section minus the inflection point offset of the two ends, that is, *l*−*S*_0_ = *l*. In addition, the residual deformation is calculated using the equivalent mining thickness *m*'.

When the collapsed area of the goaf is activated, assuming that all the voids of the fractured rock mass are fully compacted, the maximum deformation value of the surface caused by single-layer mining will not exceed the thickness *m*, and the ultimate subsidence coefficient will be 1.0 [17]. The equivalent mining thickness *m*' of residual deformation is as follows.

$$m^{'}=m(1-q)$$ (8)

Based on Eq (7) and Eq (8), the residual deformation caused by the equivalent mining thickness of the collapsed area in the goaf can be obtained.

$$W_{c}(s)=\frac{m^{'}\cos\alpha^{'}}{r}\int_{0}^{l}\exp\{-\frac{\pi {\left[x-s-h\cot\theta_{0}(\alpha ,x)\right]}^{2}}{r^{2}}\}dx$$ (9)

Then, residual inclination deformation *i*_*c*_(*s*), residual curvature deformation *k*_*c*_(*s*), residual horizontal displacement *U*_*c*_(*s*), and residual horizontal deformation *ε*(*s*) can be obtained.

$$\begin{array}{l} i_{c}(s)=\frac{dW_{c}(s)}{ds}=\frac{\pi m^{'}\cos\alpha^{'}}{r^{3}}\times \\ \;\int_{0}^{l}\exp\{-\frac{\pi {\left[x-s-h\cot\theta_{0}(\alpha ,x)\right]}^{2}}{r^{2}}\}(2x-2s-2h\cot\theta_{0}(\alpha ,x))dx \end{array}$$ (10)

$$\begin{array}{l} k_{c}(s)=\frac{di_{c}(s)}{ds}=\frac{\pi^{2}m^{'}\cos\alpha^{'}}{r^{5}}\times \\ \;\int_{0}^{l}\exp\{-\frac{\pi {\left[x-s-h\cot\theta_{0}(\alpha ,x)\right]}^{2}}{r^{2}}\}{\left[2x-2s-2h\cot\theta_{0}(\alpha ,x)\right]}^{2}dx\\ \;-\frac{2\pi m^{'}\cos\alpha^{'}}{r^{3}}\int_{0}^{l}\exp\{-\frac{\pi {\left[x-s-h\cot\theta_{0}(\alpha ,x)\right]}^{2}}{r^{2}}\}dx \end{array}$$ (11)

$$\begin{array}{l} U_{c}(s)=bri_{c}(s)=br\frac{dW_{c}(s)}{ds}=\frac{b\pi m^{'}\cos\alpha^{'}}{r^{2}}\times \\ \;\int_{0}^{l}\exp\{-\frac{\pi {\left[x-s-h\cot\theta_{0}(\alpha ,x)\right]}^{2}}{r^{2}}\}(2x-2s-2h\cot\theta_{0}(\alpha ,x))dx \end{array}$$ (12)

$$\begin{array}{l} \varepsilon (s)=\frac{dU_{c}(s)}{ds}=brk_{c}(s)=\frac{b\pi^{2}m^{'}\cos\alpha^{'}}{r^{4}}\times \\ \;\int_{0}^{l}\exp\{-\frac{\pi {\left[x-s-h\cot\theta_{0}(\alpha ,x)\right]}^{2}}{r^{2}}\}{\left[2x-2s-2h\cot\theta_{0}(\alpha ,x)\right]}^{2}dx\\ \;-\frac{2b\pi m^{'}\cos\alpha^{'}}{r^{2}}\int_{0}^{l}\exp\{-\frac{\pi {\left[x-s-h\cot\theta_{0}(\alpha ,x)\right]}^{2}}{r^{2}}\}dx \end{array}$$ (13)

where *b* is the coefficient of horizontal movement, which is the ratio of the maximum horizontal movement value *U*_max_ to the measured maximum surface subsidence value *W*_max_.

### Dynamic prediction model of the residual subsidence

**Fig 4:**
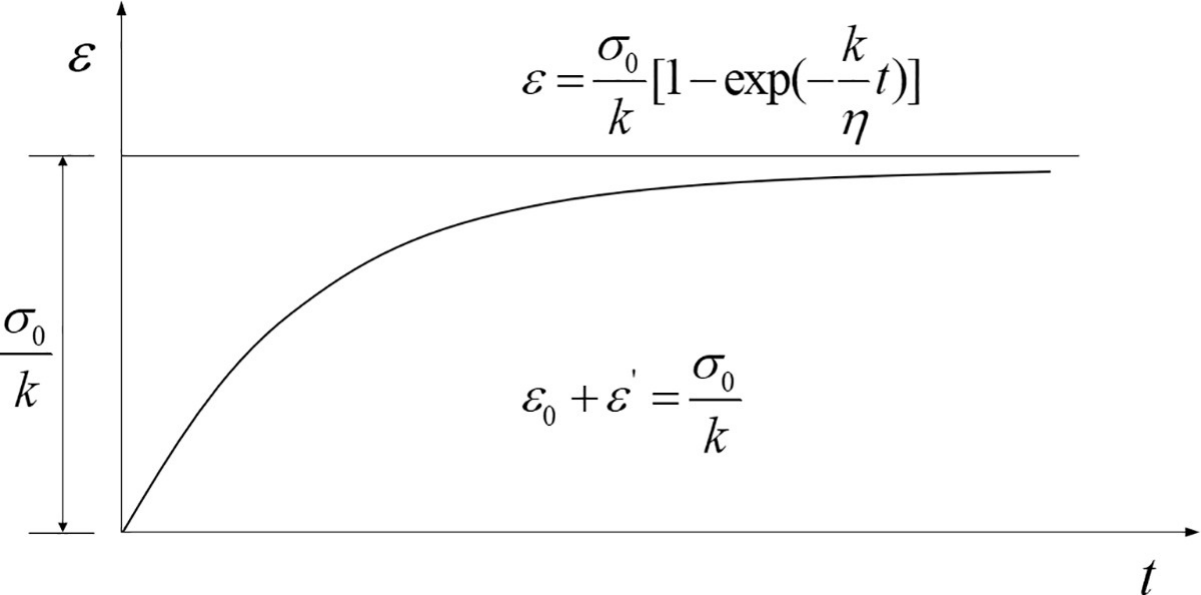
Kelvin creep curve.

The predicted residual deformation value of the above models is the potential maximum residual value. The mining voids are fully compacted after the collapsed area is activated. Therefore, the potential maximum residual subsidence value can be obtained by the surface residual subsidence curve function of Eq (9). The voids in the fractured rock mass in the collapsed area decrease over time. The strain of the rock mass is increasing, and finally it tends to be constant. In the dynamic prediction of the surface residual subsidence, the surface subsidence gradually increases, and finally, it tends to the potential maximum residual subsidence value. In rock mechanics, the compaction process of the rock mass in the collapsed area can be regarded as the creep process of rock, which can be described by Kelvin model [18]. Kelvin creep model can be seen in Fig 4.

Kelvin model is a kind of viscoelastic body, and the mechanical model is as follows:

(1) Constitutive equation

The relation between stress and strain.

$$\{\begin{array}{c} \sigma =\sigma_{1}+\sigma_{2}\\ \varepsilon =\varepsilon_{1}+\varepsilon_{2}\\ \sigma_{1}=k\varepsilon_{1}+k\varepsilon \\ \sigma_{2}=k\varepsilon_{1}^{'}+k\varepsilon^{'} \end{array}$$ (14)

Then, the constitutive equation of Kelvin model can be obtained by Eq (14).

$$\sigma =k\varepsilon +\eta \varepsilon^{'}$$ (15)

(2) Creep equation

If an invariant stress *σ*_0_ is applied at *t* = 0, the constitutive equation can be expressed as follows.

$$\{\begin{array}{c} \sigma_{0}=k\varepsilon +\eta \frac{d\varepsilon }{dt}\\ \frac{d\varepsilon }{dt}+\frac{k}{\eta }=\frac{1}{\eta }\sigma_{0} \end{array}$$ (16)

Then, the following result can be obtained.

$$\varepsilon =\frac{\sigma_{0}}{k}[1-\exp(–\frac{k}{\eta }t)]$$ (17)

(3) Residual subsidence dynamic prediction model

From the creep equation, *ε* = *σ*_0_/*k* tends to be constant when *t*→∞. In fact, in the process of rock creep, the voids of the fractured rock mass in the collapsed area of the goaf are compacted, and the residual subsidence value tends to the maximum potential subsidence *W*_*c*_(*s*). The parameter *k*/*η* in the creep equation can be integrated into the parameter *μ*, which is affected by time. The dynamic prediction model of surface residual subsidence can be written as follows.

$$W_{c}(s,t)=W_{c}(s)[1-\exp(‐\mu t)]$$ (18)

## Reliability analysis of the prediction model

### Engineering background

**Fig 5:**
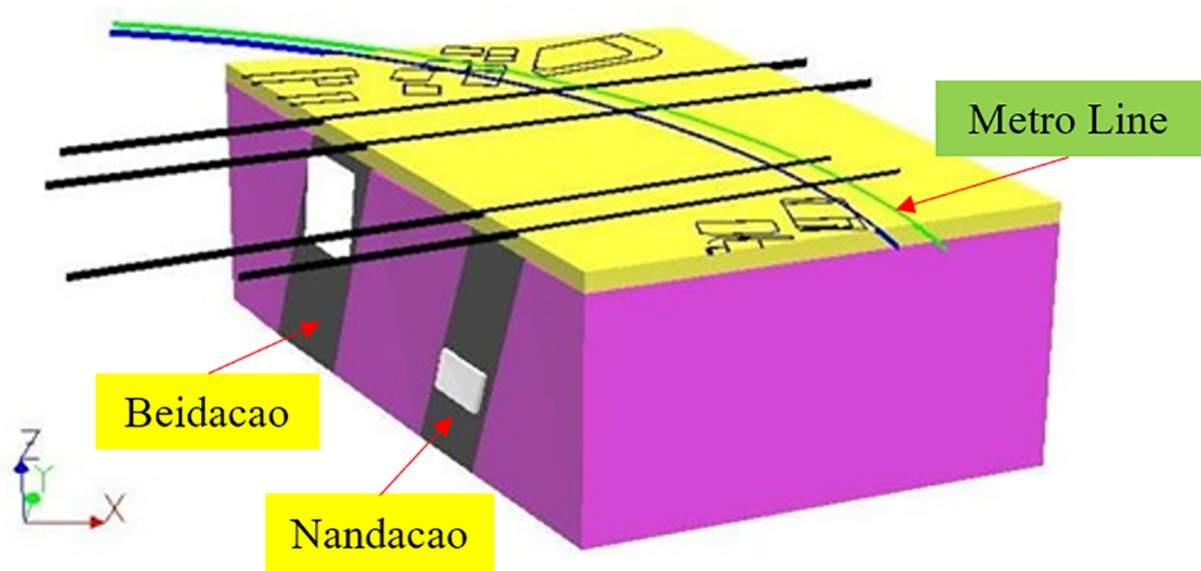
Diagram of the mining horizon of the Liudaowan coal mine.

Urumqi Metro Line–1 starts from Santunbei Station in the south and ends at Diwopu Airport Station in the north. The metro line goes through the Liudaowan coal mine, and the diagram of the mining horizon is shown in Fig 5. The Liudaowan coal mine of the Urumqi coalfield is in the south of the Dzungaria Basin of China, is at the south edge of Urumqi city and is adjacent to the Xishan fault group. The Liudaowan mine has been mined since 1988, and its main mining coal seam is a steeply inclined extra-thick coal seam in Nandacao (B1+2) and Beidacao (B3+6) with a thickness of 45~50 m and an angle of 70°~80°. The Nandacao mining warehouse artificial caving collapse occurred in 1998, and two beaded groove-shaped collapses in the ground were eventually observed. Two large-scale V-shaped collapsed grooves are located on both sides of the giant rock pillar between the B1+2 and B3+6 coal seams [19].

When the metro is built above the collapsed area, the collapsed area will generate residual deformation under the disturbance of external forces and cause uneven ground settlement, which in turn affects the normal use of the metro structure and ground buildings. Therefore, it is necessary to carry out research on the residual deformation of collapsed areas to analyze the problem of foundation stability.

### Theoretical model verification and dynamic prediction

#### Static residual deformation verification

To verify the accuracy of the residual deformation prediction model based on the mining influence propagation angle, the monitoring data of the Beidacao and the Nandacao of the Liudaowan coal mine in Urumqi are chosen as samples [20]. For the goaf, the average depth of the 750 m horizontal coal seam in Beidacao is 70 m and the average depth of the 720 m horizontal coal seam in Nandacao is 100 m. The dip angle of the coal seam is almost 75°, the average coal thickness is 40 m, the mining height is approximately 45 m and the mining length is approximately 200 m. The subsidence coefficient can be obtained as *q* = 0.98 based on the monitoring data. The basic parameters of the surface deformation of the Liudaowan coal mine in Urumqi are listed in Table 1. The predicted values of surface residual deformation of the subsidence area for Beidacao and Nandacao of the Liudaowan coal mine are calculated from Eq (9) to Eq (13). The predicted values and monitoring values are shown in Table 2.

**Table 1:** Basic parameters of the surface deformation prediction of the Liudaowan coal mine.

Parameter	Beidacao	Nandacao
Mining height *M*/mm	45000	45000
Average depth *h*/m	70	100
Subsidence coefficient *q*	0.98	0.98
Tangent of main influence angle tan*β*	2.0	2.0
Inflection point offset *S*_0_/m	0	0
Horizontal movement coefficient *b*	0.2	0.2

**Table 2:** Residual deformation comparison of the monitoring and prediction of the Nandacao and Beidacao goafs.

Name		Residual subsidence	Residual inclination deformation	Residual curvature deformation	Residual horizontal displacement	Residual horizontal deformation
		*W*_*c*_(*s*)(mm)	*i*_*c*_(*s*)(mm⋅m^−1^)	*k*_*c*_(*s*)(mm⋅m^−2^)	*U*_*c*_(*s*)(mm)	*ε*(*s*)(mm⋅m^−1^)
Beidacao	Maximum measured value	614	15.7	1.938	158	21.45
	Maximum predicted value	593	14.96	2.02	149.6	20.2
	Relative error	3%	5%	-4%	5%	6%
Nandacao	Maximum measured value	609	15.6	1.945	156	21.68
	Maximum predicted value	583	14.5	2.027	145	20.27
	Relative error	4%	7%	-4%	7%	6%

For Beidacao, the highest value of relative error is only 6%, that is, the difference between the measured values and the predicted values is small. For Nandacao, the values of relative error are in the range of ±7%, and the maximum predicted values are close to the maximum measured values. It can be said that the prediction model can accurately predict the residual deformation of the goaf.

#### Dynamic residual subsidence prediction

**Fig 6:**
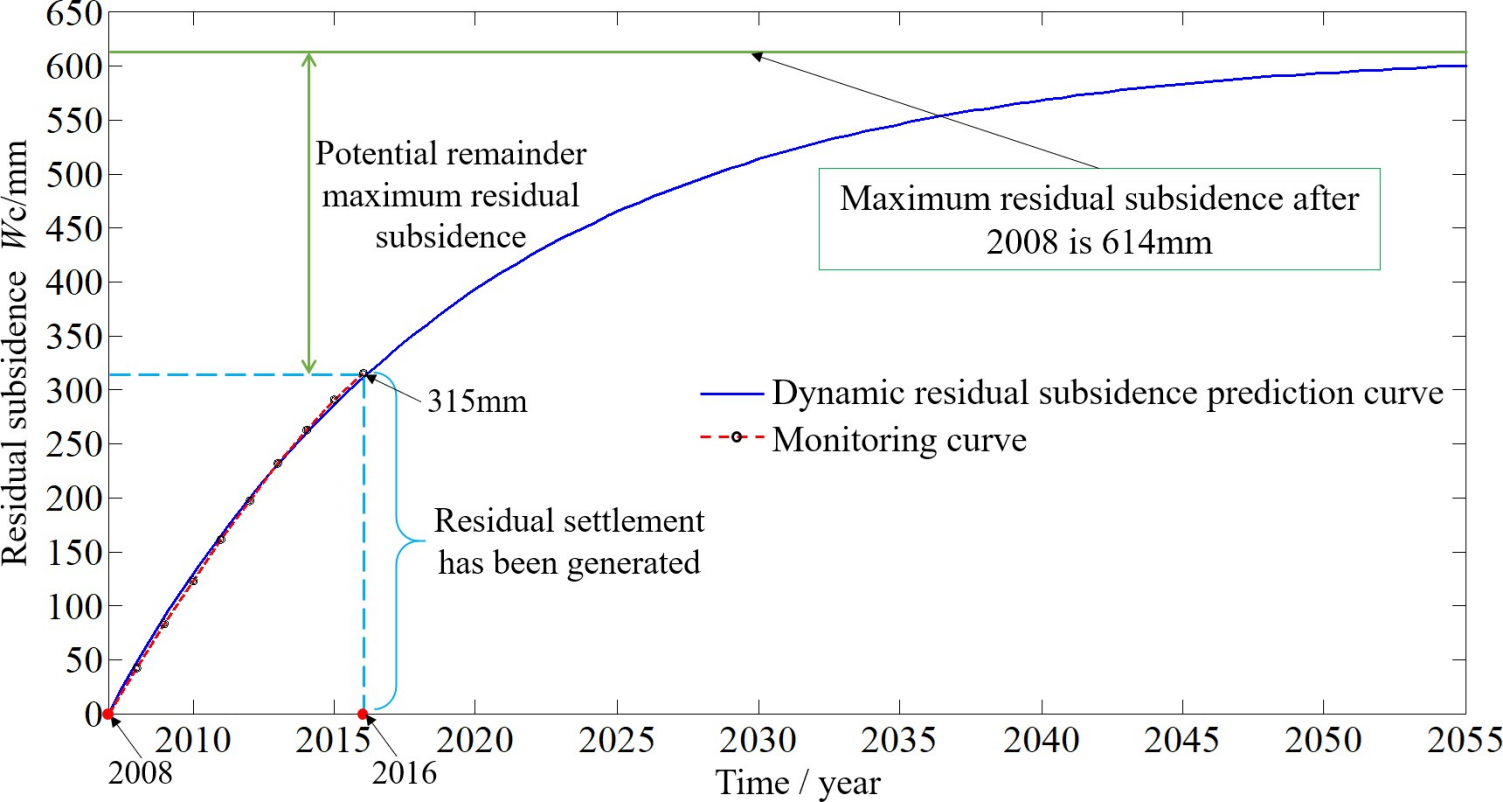
Comparison of the monitoring value and dynamic prediction value of the residual subsidence in the Beidacao goaf.

To guide the subsequent metro construction, the crossing section of the designed metro line is selected as an example to analyze the dynamic residual subsidence. The residual deformation data for the Beidacao goaf of the Liudaowan coal mine in 2008–2016 are applied to the residual subsidence dynamic prediction model (Eq (18)) for curve fitting. Then, the parameter *μ* and maximum measured value of residual subsidence *W*_*c*_(*s*) can be obtained, whose values are 0.0784 and 614 mm, respectively. The monitoring value and dynamic prediction value of the residual subsidence in the Beidacao goaf are shown in Fig 6.

As shown in Fig 6, the fitted values (predicted data) from 2008 to 2016 are close to the monitoring data. The average relative error is only 4.1%. The maximum and minimum relative errors are 9.5% and 0.3%, respectively. It can be considered that the predicted results can accurately reflect the actual situation. At the end of 2016, the residual deformation value calculated by the prediction model is 303 mm, which is close to the measured residual deformation value of 299 mm. The model can effectively describe the dynamic process of residual subsidence, which indicates that the prediction model can be used as the theoretical basis for residual subsidence. The dynamic prediction curve can provide guidance for subsequent metro construction.

#### Numerical simulation verification

**Fig 7:**
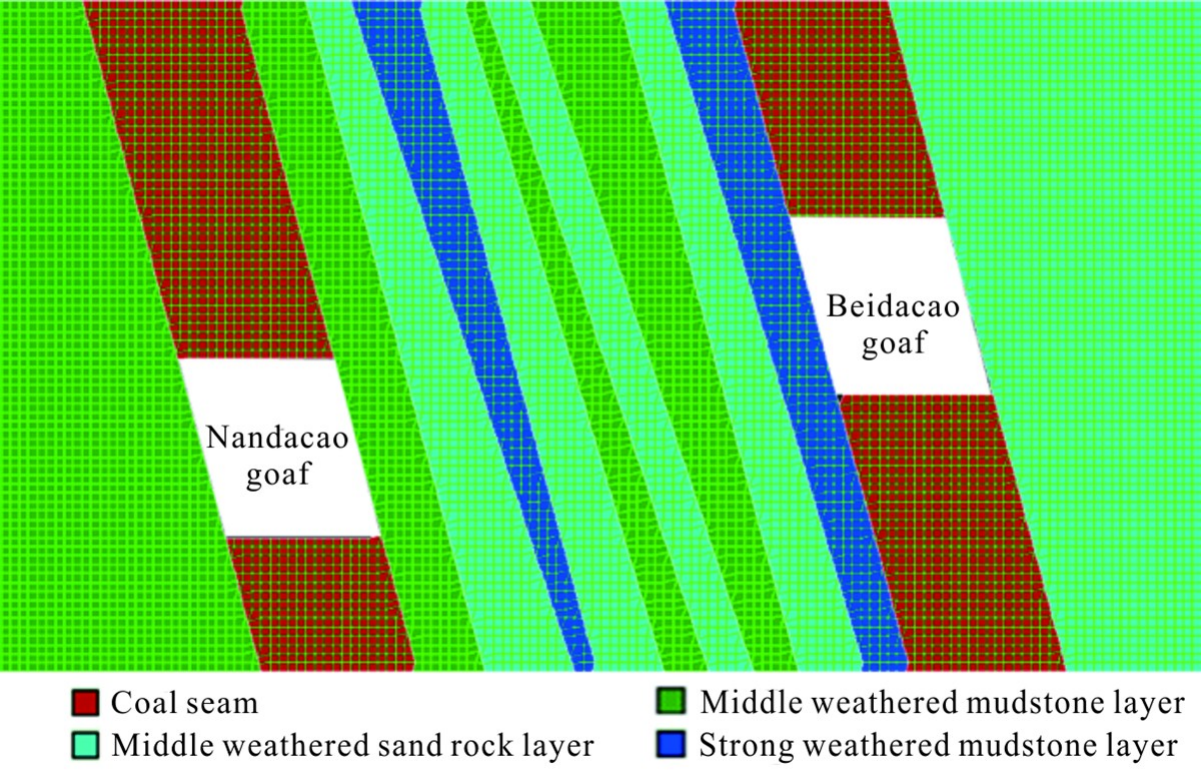
Numerical model figure of the Nandacao and Beidacao seams after excavation.

According to the general situation for the Nandacao and Beidacao goafs of the Liudaowan coal mine in Urumqi and the investigation report of Urumqi Metro Line 1 [21], the numerical model for the Nandacao and Beidacao coal seams after excavation is established, which can be seen in Fig 7. The site rocks are mainly four types of rock groups: the middle weathered mudstone layer, middle weathered sand rock layer, strong weathered mudstone layer and coal seam. These materials are defined by the Mohr-Coulomb model in the calculation process. Only the broken rock mass in the collapsed area is defined as the Burgers-Creep constitutive model. The coal and rock mechanical parameters are shown in Table 3. The parameters of the Burgers-Creep constitutive model are inverted based on the measured data. The numerical simulation mechanical parameters of the Burgers creep model are listed in Table 4.

**Table 3:** Mechanical parameters.

Name	Density (kg/m^3^)	Elastic modulus (GPa)	Poisson ratio	Cohesion (kPa)	Internal friction angle (°)
Strong weathered mudstone layer	2420	1.7	0.32	9.2	25.2
Middle weathered mudstone layer	2550	3.5	0.28	145.8	34.3
Middle weathered sand rock layer	2540	5.1	0.25	124.3	29.7
Coal seam	1560	1.3	0.28	61.2	31.1
Sand gravel fillings	1720	0.5	0.28	0	21.3
Collapsed coal body	1230	0.3	0.31	0	15.3

**Table 4:** Mechanical parameters of the Burgers creep model.

*η*_*k*_ (GPa⋅s)	*η*_*m*_ (GPa⋅s)	*G*_*k*_(MPa)	*G*_*m*_(MPa)
38.2	85.4	325.0	1.5

After the excavation is completed, collapse occurs in the Nandacao and Beidacao goafs, and two collapse pits are formed on the surface, resulting in subsidence of the surface. Then, the goaf is filled with broken rock masses, which are loose and not dense. When the old goaf is activated, the gaps of these broken rocks are continuously compacted, resulting in residual deformation on the surface. Because Urumqi Metro Line 1 passes through the Nandacao and Beidacao goafs, it is of great importance to analyze the residual deformation of the goaf before metro excavation.

**Fig 8:**
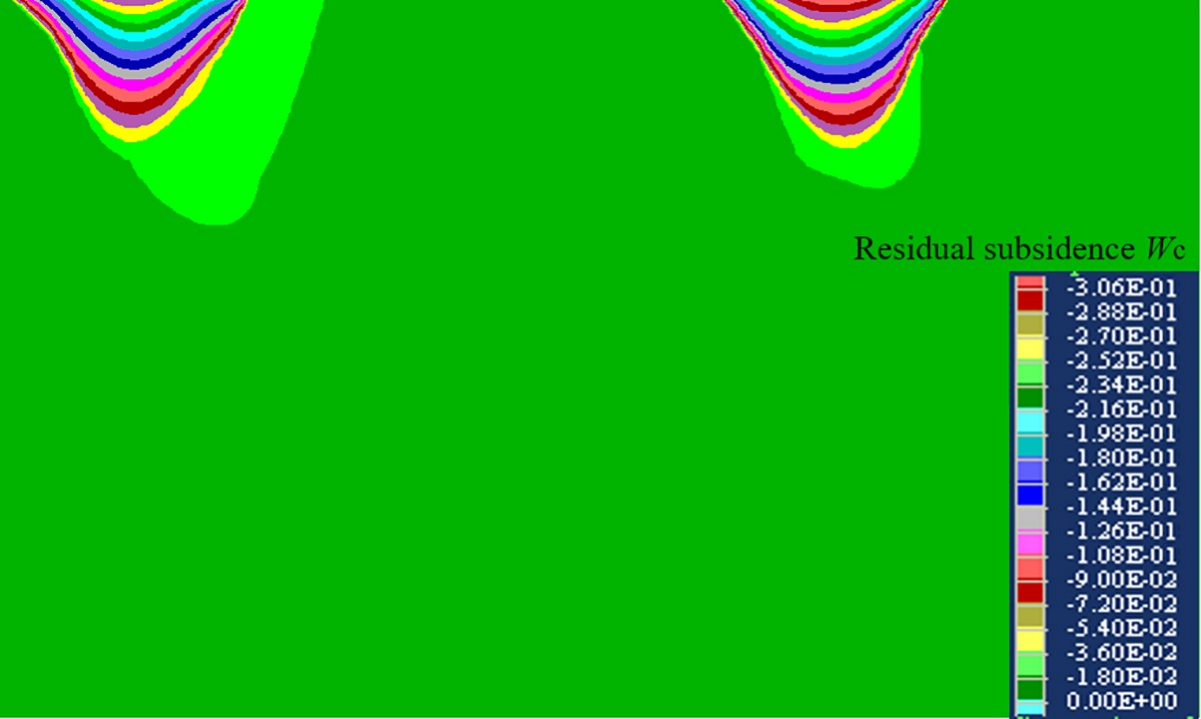
Calculation result of the residual subsidence numerical model.

**Fig 9:**
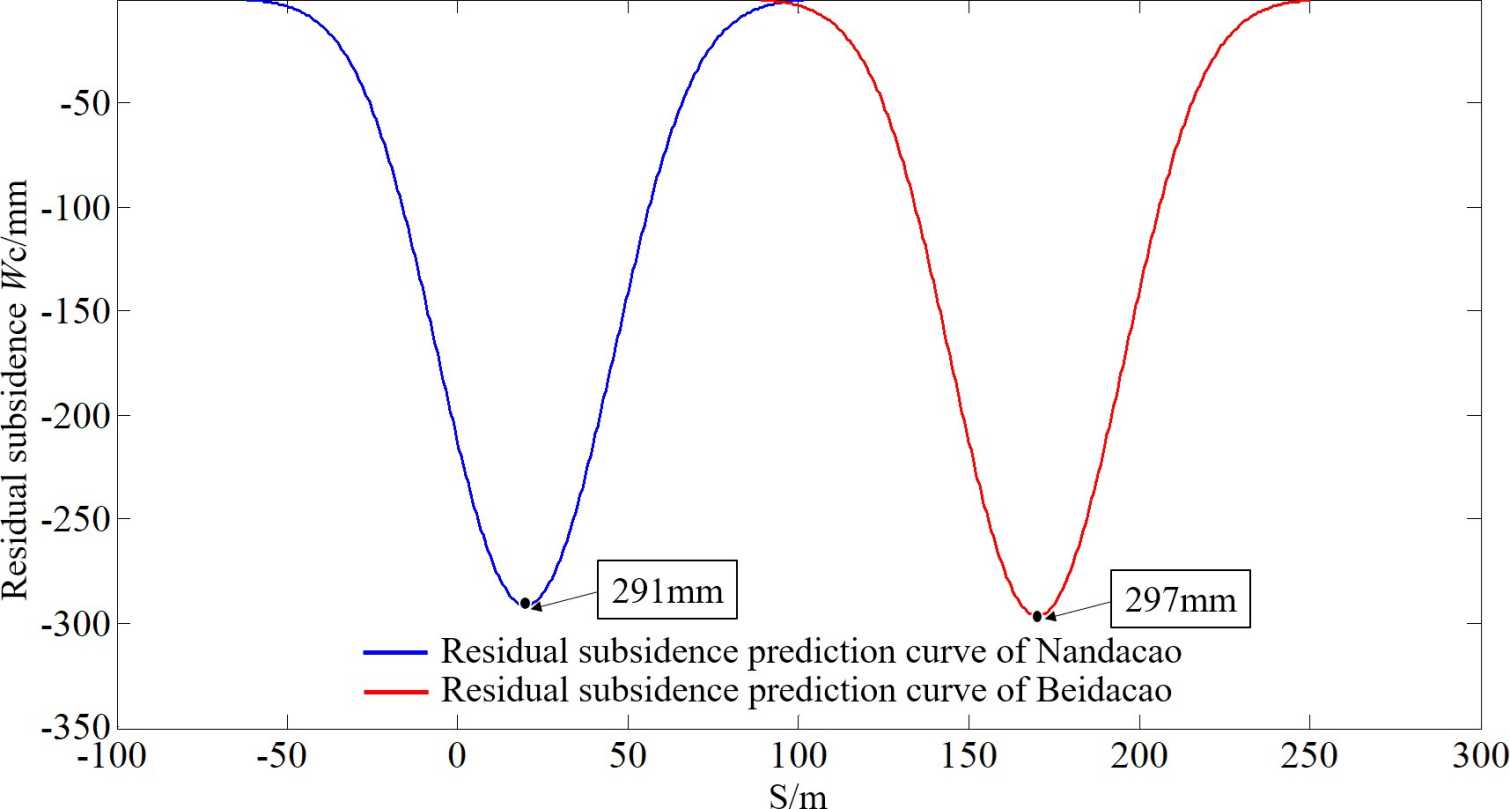
Calculation result of the prediction model for residual subsidence.

The numerical simulation result of the residual subsidence before the metro excavation can be seen in Fig 8. The calculation results of the prediction model (Eq (9)) of the residual subsidence are shown in Fig 9.

As shown in Fig 8, the maximum residual subsidence of the Nandacao and Beidacao goafs is 288~306 mm according to the numerical simulation. Based on the residual subsidence prediction model (Eq (9)), the maximum residual subsidences of the Nandacao and Beidacao goafs are 291 mm and 297 mm, respectively. The numerical simulation results are close to those of the prediction model. The shape of the prediction model curve of the residual subsidence is consistent with the numerical simulation, which has a concave shape. The reliability of the prediction results is verified again.

## Conclusions

In this paper, the mining influence propagation law in the steeply inclined extra-thick coal seam is analyzed, and the variable mining influence propagation angle is used to describe the special form of the rock movement. A subsidence prediction model based on the variable mining influence propagation angle is constructed according to the modified parameters in the traditional probability integral model. Considering that the residual deformation is due to the compaction of the broken rock mass space in the caving zone, Kelvin model is introduced to study the creep characteristics of the old goaf, and the dynamic prediction function of residual subsidence is constructed.

Finally, the prediction models are applied for residual subsidence prediction. The results of the case study show that the predicted maximum value of the surface residual deformation is relatively close to the measured maximum value. Therefore, the residual deformation prediction model based on the mining influence propagation angle is a promising method for the evaluation of the foundation stability of engineering projects crossing a goaf.
